# Scaling Up Improved Inpatient Treatment of Severe Malnutrition: Key Factors and Experiences From South Africa, Bolivia, Malawi, and Ghana

**DOI:** 10.9745/GHSP-D-21-00411

**Published:** 2022-04-28

**Authors:** Shuaib Kauchali, Thandi Puoane, Ana Maria Aguilar, Sylvester Kathumba, Alice Nkoroi, Reginald Annan, Sunhea Choi, Alan Jackson, Ann Ashworth

**Affiliations:** a Nelson Mandela University, University Way, Summerstrand, Gqeberha, South Africa.; b National Department of Health, Pretoria, South Africa.; c School of Public Health, University of the Western Cape, Bellville, South Africa.; d Instituto de Investigación en Salud y Desarrollo, Universidad Mayor de San Andrés, La Paz, Bolivia.; e Ministry of Health, Lilongwe, Malawi.; f Food and Nutrition Technical Assistance Project (FANTA)/FHI360, Washington, DC, USA.; g Department of Biochemistry and Biotechnology, Kwame Nkrumah University of Science and Technology, Kumasi, Ghana.; h Human Development and Health, Faculty of Medicine, University of Southampton, Southampton, United Kingdom.; i International Malnutrition Task Force of the International Union of Nutritional Sciences, London, United Kingdom.; j Department of Population Health, London School of Hygiene & Tropical Medicine, London, United Kingdom.

## Abstract

Severe acute malnutrition (SAM) can have high mortality, especially in very ill children treated in the hospital. Many medical and nursing schools do not adequately, if at all, teach how to manage children with SAM. There is a dearth of experienced practitioners and trainers to serve as exemplars of good practice or participate in capacity development. We consider 4 country studies of scaling up implementation of WHO guidelines for improving the inpatient management of SAM within under-resourced public sector health services in South Africa, Bolivia, Malawi, and Ghana. Drawing on implementation reports, qualitative and quantitative data from our research, prospective and retrospective data collection, self-reflection, and our shared experiences, we review our capacity-building approaches for improving quality of care, implementation effectiveness, and lessons learned. These country studies provide important evidence that improved inpatient management of SAM is scalable in routine health services and scalability is achievable within different contexts and health systems. Effectiveness in reducing inpatient SAM deaths appears to be retained at scale.

The country studies show evidence of impact on mortality early in the implementation and scaling-up process. However, it took many years to build workforce capacity, establish monitoring and mentoring procedures, and institutionalize the guidelines within health systems. Key features for success included collaborations to build capacity and undertake operational research and advocacy for guideline adoption; specialist teams to mentor and build confidence and competency through supportive supervision; and political commitment and administrative policies for sustainability. For frontline staff to be confident in their ability to deliver appropriate care competently, an enabling environment and supportive policies and processes are needed at all levels of the health system.

## Abstract (precis)

We report lessons learned in 4 countries from scaling up the implementation of World Health Organization guidelines on inpatient management of severe acute malnutrition within routine health services. We provide evidence that implementation is achievable at scale within different contexts and health systems.

## Teaser

**Key Findings:**

- We report our “how-to” approaches and lessons learned from scaling up implementation of the World Health Organization (WHO) guidelines on inpatient treatment of severe acute malnutrition within routine health services in 4 countries.
- Substantial improvements in survival were evident within a few months of guideline implementation, but it took many years to improve treatment practices at scale.
- We show that the WHO guidelines are scalable to a national level, and their effectiveness in improving survival appears to be retained at scale. Key features for success include collaborations to build capacity and undertake operational research and advocacy for guideline adoption; specialist teams to mentor and build confidence and competency through supportive supervision; and political commitment and administrative policies for sustainability.

**Key Implications:**

- Policy makers, health professionals, and their national societies should bring together all the talent available and establish alliances such as communities of practice to implement and scale up WHO malnutrition treatment guidelines and use new opportunities such as eLearning to build workforce capacity.
- Health educators should review medical and nursing curricula and re-align pre-service training so that it is fit-for-purpose and competency-based for effective practice.

Resumen en español al final del artículo.

## INTRODUCTION

Significant progress has been made in preventing and treating severe acute malnutrition (SAM) since 2007 when United Nations agencies recommended adopting an integrated approach, known as community-based management of acute malnutrition (CMAM)^1^. This approach includes the active identification of children at risk through community-based screening and treatment with therapeutic foods to restore deficits in weight, which can be very effective in those without complications.^2^ Children who are more seriously ill with complications require a higher level of care as inpatients, and CMAM also includes this aspect. The World Health Organization (WHO), UNICEF, and other partners have developed guidelines for identifying and managing SAM that follow the same underlying principles. The WHO guidelines for inpatient management are centered on 10 steps.^3^^,^^4^ When applied with diligence, in context, they have been found to be both efficacious and effective, with case fatality rates less than 5% even in resource-constrained settings.^2^^,^^5^^–^^8^ Even so, taking all aspects of integrated care to scale in the development context has been challenging, being vulnerable to any weakness in health delivery systems. This is especially so for inpatient management of children with SAM and complications, given the attention to detail required for the successful care of very sick children and the counterintuitive nature of some aspects of care in relation to standard pediatric approaches. In this article, we focus on inpatient management, given the higher risk of death in these complicated cases.

Before the coronavirus disease (COVID-19) pandemic, an estimated 13.6 million children were considered to be severely wasted.^9^ Scaling up proven effective treatment of SAM could reduce child mortality substantially in low- and middle-income countries (LMICs).^5^ The need for action is acute, and many nations face increased hunger and malnutrition from food insecurity, disruption of child health services, and loss of livelihoods associated with COVID-19.^10^ For example, 52% of 90 countries surveyed by WHO in May 2020 reported partial or severe disruption of essential services for sick or malnourished children during the pandemic, with likely adverse consequences for survival.^11^

Scaling up proven effective treatment of SAM could reduce child mortality substantially in LMICs.

Although SAM often arises in humanitarian emergencies, most cases occur in nonemergency situations where public health services are chronically underfunded. The great challenge is how to scale up within routine health services with limited resources, particularly regarding capacity development and integration of quality of care into national plans and policies.

In the 1970s, 2 authors (AA and AJ) helped scale up inpatient treatment guidelines for SAM in the Caribbean and establish pre-service and in-service training.^8^ Scaling up was relatively easy given the geographic size of these island nations and their regional cohesion. However, there are few examples of successful scale-up in more challenging contexts of most LMICs.

We invited implementers in South Africa, Bolivia, and Malawi, where WHO malnutrition guidelines have been scaled up successfully, to describe their implementation processes, provide effectiveness data, and relate their experiences and lessons learned for this article. In Ghana, we invited an implementer of CMAM and implementers of an innovative, scalable eLearning approach to capacity building to join.

In this descriptive account, we consider experiences in South Africa, Bolivia, Malawi, and Ghana of scaling up implementation of WHO inpatient guidelines within routine health services. Eight of the 9 authors were directly involved in guideline implementation in at least 1 of the 4 country studies. We have drawn on our contemporaneous implementation reports, qualitative and quantitative data from our research papers,^12^^–^^26^ and our prospective and retrospective data collection. Over several years, 7 of the authors had met and participated in international symposia and workshops where we presented our findings from South Africa, Bolivia, and Ghana. These opportunities facilitated self-reflection and sharing of experiences, many of which we have captured in this article.

South Africa and Bolivia started in just 1 hospital and have scaled up nationwide. In Malawi and Ghana, scaling up of WHO inpatient guidelines was linked to CMAM. A major hurdle to scaling up in LMICs is the lack of skilled and experienced professionals for capacity development. The country studies illustrate how this hurdle was tackled, the effectiveness of implementation, lessons learned, and key factors for success.

We use the WHO ExpandNet definition of scale-up^27^:

*Deliberate efforts to increase the impact of innovations successfully tested in pilot or experimental projects so as to benefit more people and to foster policy and programme development on a lasting basis.*

## SOUTH AFRICA COUNTRY STUDY

South Africa, a middle-income country with a population of 60 million, has 2 parallel, unequal health systems. The public system serves around 80% of the population, and the private health sector employs about 80% of the country's doctors. Scaling up implementation of improved inpatient management of SAM took 18 years, beginning in 1998 when case fatality rates (CFR) of 30%–50% were not unusual. Since 2009, national inpatient fatality rates for SAM have been reported annually through the Child Healthcare Problem Identification Programme.^12^ The Figure shows that from 2009 to 2021, the average CFR fell from 19.2% to 7.0%. Although adherence to the 10 steps was not routinely measured, the decline suggests that improved inpatient case management has contributed to the decreased CFR.^13^

**FIGURE:**
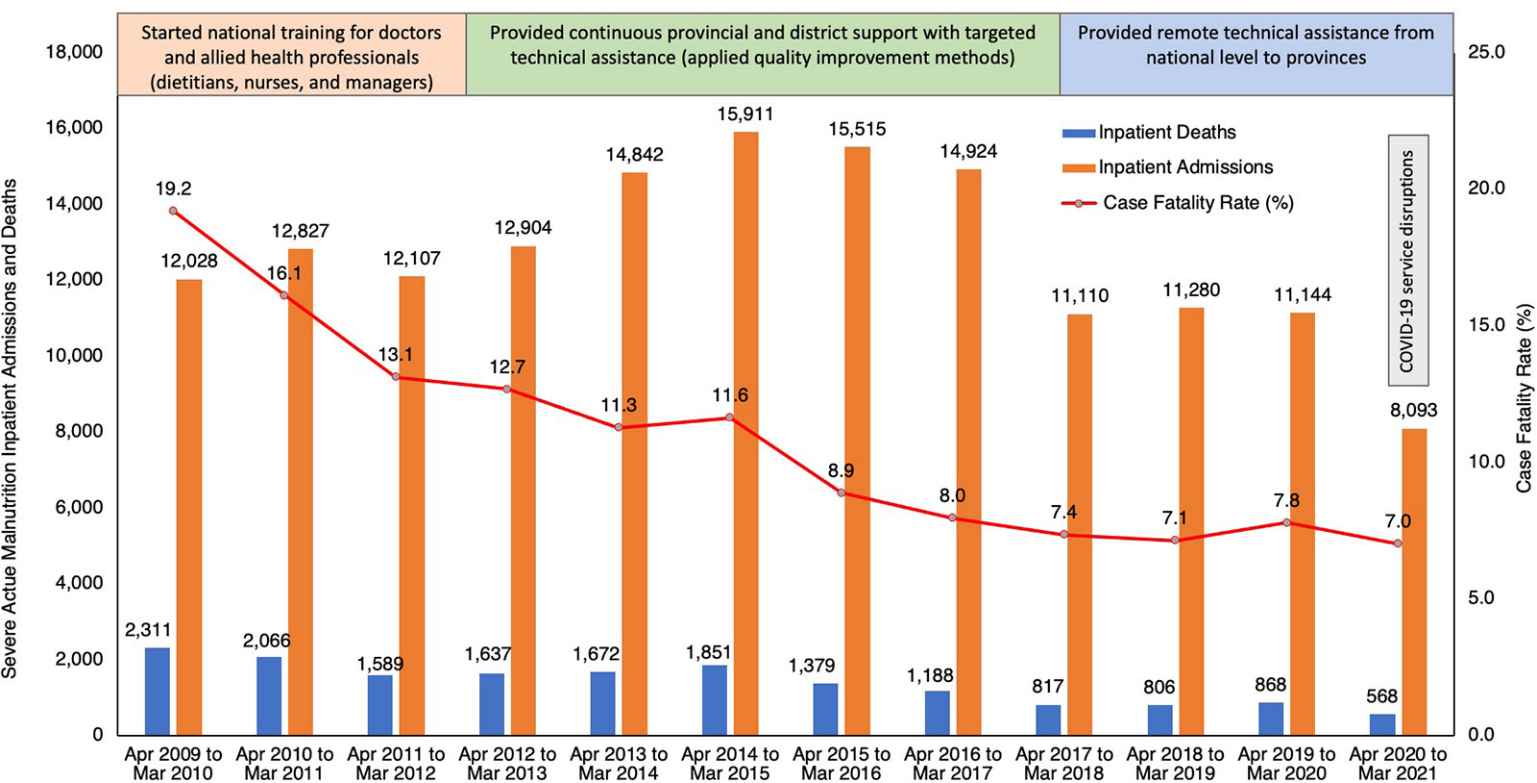
Severe Acute Malnutrition Inpatient Admissions, Deaths, and Case Fatality Rates in South Africa, 2009–2021^a,b^ ^a^Source: District Health Information System, abstracted January 2022. ^b^Upper boxes show a timeline for training and support during national scale-up.

From 2009 to 2021 in South Africa, CFR fell from 19.2% to 7.0%, suggesting that scaling up implementation of improved inpatient management of SAM contributed to the decrease.

Box 1 gives an overview of the events that led to scaling up nationwide.

BOX 1 **Capacity Building to Reduce Inpatient Deaths From Severe Acute Malnutrition in South Africa**

The South Africa program began in Mary Theresa Hospital in the former Mount Frere health district, within a health system poorly resourced after years of apartheid neglect. The program grew from formative researchby pediatric ward staff aided by external facilitators as part of a pilot model district Integrated Nutrition Project developed by the Health Systems Trust, the Eastern Cape Department of Health (DOH), and the University of the Western Cape.

**Formative research:** Mary Theresa Hospital had a case fatality rate (CFR) of 50%, and none of the 10 steps was practiced. Children were not fed at night or kept warm, those with diarrhea received intravenous fluid indiscriminately and were not monitored, electrolyte and micronutrient deficiencies were not corrected, antibiotics were not given routinely, and feeds were inappropriate. There was lack of knowledge and motivation among staff, overcrowding, and inadequate supplies. The main causes of death were considered to be hypoglycemia and hypothermia, cardiac failure from overhydration and electrolyte imbalance, and untreated infections.

**1998–Methods used to introduce quality improvement:** Two 2-day workshops were held for pediatric ward staff at Mary Theresa and Sipetu hospitals and key administrators to explain the physiological changes in severe acute malnutrition (SAM) and their implications for treatment, review inadequacies in patient care (key factor 1), make action plans for implementing the 10 steps, identify logistical difficulties and possible solutions, and review progress. For 5 months, a facilitator made monthly follow-up visits, during which they monitored performance and impact indicators, observed ward procedures, checked patient records for completeness and correctness, and provided feedback and coaching to address specific issues.

**2000–Impact at the first 2 hospitals:** Despite HIV comorbidity, CFR fell from 50% to 21% at Mary Theresa hospital and from 28% to 18% at Sipetu hospital.^14^^,^^16^ Motivation was high, and staff were eager to improve further.

**2000–Scaling up within the region:** All 11 district hospitals had participated in training. A nutrition coordinator made monthly supervision/monitoring visits. At quarterly death review meetings, 2 nominees from each hospital presented a case for discussion. CFRs fell by one-third on average from a median of 28%, and 2 hospitals achieved CFRs of 6%.

**2002–Scaling up in Eastern Cape and other provinces:** Twenty-five hospitals had been trained. The Eastern Cape DOH took over responsibility from external facilitators for replication in the province, and nurses comprised the training team. In 2008, the National DOH took responsibility for scaling up in the rest of the country12,18 (refer to the key factors and Discussion section). National scale-up was designed to integrate policy makers, provincial managers (enablers), technical assistance specialists, and district and facility implementers (doers). The training targeted district hospital staff where most of the SAM admissions occurred. Scaling up was achieved by 2016/2017, most of which occurred after 2011.

In the following sections, we provide further details about the training, monitoring, and support activities and list some key factors that facilitated change in quality of care drawn from implementation research in the Eastern Cape and national experience.^14^^–^^20^ Later in the article, we list additional key factors relevant to Bolivia, Malawi, and Ghana.

### Key Factor 1: Participatory Ethos

In Eastern Cape, a participatory approach to capacity building was planned at the outset.^14^^,^^15^ First, pediatric ward staff were equipped with research skills to assess and analyze the situation on their ward. Calculating CFRs from ward registers revealed the enormity of deaths, and comparing treatment practices against the 10 steps enabled staff to identify inadequacies in patient care and plan actions. Participation engendered ownership, motivation to improve care, and commitment.

### Key Factor 2: Involvement of Hospital Managers and District Health Personnel

Ward staff implemented many of the 10 steps themselves, including moving from 3 daytime meals to 8 3-hourly specially prepared feeds. Some difficulties, such as unheated wards from electricity outages, drug supplies, and broken sinks, required action by hospital and nursing service managers, pharmacy department, and the maintenance unit. Provision of the electrolyte/mineral solution required negotiation with provincial personnel. Involvement of key managerial staff in the training workshops aided implementation. In Mount Frere district, hospital policy was changed to allow mothers to stay overnight to help feed and keep children warm and to cease rotation of nurses from the pediatric ward to other wards.

### Key Factor 3: Data Gathering for Action and Advocacy

Acquiring skills to calculate CFRs and rates of weight gain enabled staff to evaluate progress. Some plotted CFRs monthly and displayed the results. A fall in CFR was greeted with pride; a rise led to reflection and action. Nurses presented findings at academic conferences and some enrolled in distance-learning courses at the University of the Western Cape. These experiences boosted confidence and morale and provided life-enhancing opportunities that had been lacking under apartheid.

Data were also used for advocacy more widely by the University of the Western Cape. Data, showing that families of children with SAM were food-insecure and were not receiving the Child Support Grant, were used in newspaper articles, submissions to the government, and a television documentary, which prompted the Minister of Social Development to remedy the situation.^17^

Quality improvement methods at the national level used problem analysis data to identify and respond to gaps in care. For example, to reduce higher mortality at night, more professional nurses were allocated to night shifts and mothers were allowed to stay overnight to give 3 am feeds. To enable immediate feeding, the starter feed F75 was placed in the casualty department.

### Key Factor 4: Building of Local Capacity for Sustainability

Lack of local capacity meant trainings were first led by the Health Systems Trust, University of the Western Cape, and United Kingdom-based staff. Some nurses who were successful implementers became champions and were encouraged to become trainers, and a trainers' guide was developed. A district malnutrition training team was formed, which provided training to other regions of Eastern Cape. Peer trainers often gave moving testimonials of the impact of implementing the 10 steps and their personal stories of how they achieved implementation emboldened others.

Training was participatory, problem-oriented, and targeted at senior pediatric nursing staff as treatment is primarily nurse-led. Where district hospitals had only 1 or 2 doctors, the Eastern Cape Department of Health allowed the sister-in-charge to start treatment without waiting for a doctor through “standing orders,” which was lifesaving.

### Key Factor 5: Induction of Incoming Doctors and Nurses

Rapid staff turnover hindered continuity of improved care as undergraduate training of doctors and nurses about SAM was inadequate and led them to make errors.^16^ Induction of new staff and those on rotation was key to sustaining low CFRs post-training.^19^ Wall posters of the 10 steps and SAM emergency care served as useful teaching aids and reminders and are now provided to all pediatric wards in South Africa.

Induction of new staff and those on rotation was key to sustaining low CFRs post-training.

### Key Factor 6: Triage and Emergency Care

Most district hospitals had no triage, so children with SAM were not given priority in the queue, contrary to WHO guidelines. Waiting to be examined and then waiting to be sent to the ward, often totaling 7 hours, aggravated a child's condition, and hypoglycemia and dehydration were not uncommon on arrival at the ward.

Emergency care in SAM differs from that in other children and mismanagement of shock and dehydration risks fluid overload and death from cardiac failure. Such deaths are often wrongly attributed to pneumonia. A national program of Emergency Triage, Assessment, and Treatment (ETAT) began in 2011, and the 10 steps emergency protocol was incorporated. The national technical team developed 3 separate wall charts for SAM: medical emergencies (hypoglycemia, hypothermia, shock, corneal ulcers); management of children with severe illnesses; and uncomplicated cases.

### Key Factor 7: Supervision, Leadership, and Teamwork

Supervision, leadership, and teamwork differentiated hospitals that sustained improved care from those who backslid.^19^^,^^20^ Lack of supervision of junior nurses led to lax practices. Failure to explain treatment to the child's mother/caregiver led to inappropriate actions, including changing flow rates of intravenous infusions and giving additional foods. Good leadership led to supportive bidirectional communication and good teamwork, all of which improved quality of care, staff morale, and job satisfaction.

During the roll-out to provinces, the national team conducted monthly check-ins with the district and facility improvement teams to review progress and address challenges regarding clinical care and supplies. The facility teams developed action plans to remedy problems and provided feedback monthly on how these were tackled. From 2012, district clinical specialists (a special cadre hired at the district level) championed the guidelines, provided clinical supervision to frontline staff, and contributed to leadership effectiveness.^18^ CFRs continued to improve (Figure) and were sustained when national mentorship visits to hospitals (2015–2018) worked through leadership structures and targeted nursing and medical managers directly involved in SAM case management, mortality auditing, and procurement of resources. District management teams also played a significant role in providing coaching and mentoring outside national visits.^12^

Facility teams developed action plans to remedy problems and provided feedback monthly on how these problems were tackled.

### Key Factor 8: Keep It Short and Simple

Clinical audits during the early phase of the national training campaign showed most SAM deaths occurred within 24 hours of admission. To address this, the national Department of Health developed a simple message for clinicians focusing on 3 “non-negotiables” to be applied to all cases within 30 minutes of arrival to a facility: treat all cases as if they had bacterial infections (overt and covert) by starting **antibiotics** immediately; **feed** with appropriate type and quantity (triple effect: prevention/treatment of hypoglycemia/hypothermia, and electrolyte/mineral imbalance); and, keep the child with the mother and keep **warm** all the time.

## BOLIVIA COUNTRY STUDY

Bolivia, with a population of 12 million, is a middle-income country geographically at the heart of South America and has a difficult topography and rich ethnic diversity. In the last 15 years, due to favorable external trade conditions and the active role of the state, inequality and poverty have decreased and access to services has improved. Currently, the Government of Bolivia is struggling to maintain these accomplishments due to the crash in oil price exports to Brazil and Argentina and the COVID-19 pandemic.

Bolivia's public health system has a social security model. Box 2 gives an overview of the training and other events that led to scaling up WHO inpatient guidelines for SAM nationwide, which started in 2003 and took approximately 10 years.^28^ CFRs were around 25% initially and decreased quickly after training and mentoring. From 2012 onward, the inpatient CFR has been 5%–7% nationally. The initial spark was a collaboration between the Universidad Mayor de San Andrés and the London School of Hygiene & Tropical Medicine with funding from WHO.

BOX 2 **Capacity Building to Reduce Inpatient Deaths From Severe Acute Malnutrition in Bolivia**

**2003–2005–Initial phase:** This included (1) formative research; (2) Spanish translation of the World Health Organization (WHO) Training Course and training of trainers; (3) initial implementation including determining the cost of WHO-recommended treatment and obtaining approval from the health insurance system so that hospitals could be reimbursed for treatment costs; and (4) follow-up and formation of a broad partnership between academia, the Bolivian Pediatric Society, and the Ministry of Health.

Formative research^21^ at Hospital del Niño, La Paz, found that WHO guidelines were largely followed in the main pediatric ward but not in other wards or the emergency room. At Hospital Boliviano Holandés, El Alto, the guidelines were poorly followed and children were classified mostly by weight-for-age.^21^ Qualitative data indicated that mothers felt despised by staff, unhappy, and uninformed as to their child's treatment and progress.^22^ Staff felt that mothers sought care too late and were noncompliant. These 2 teaching hospitals, which had appropriate training conditions, served as the training hub during the later expansion phase.

The training, using the 6-day WHO Training Course, was held at Hospital del Niño in 2004. Forty doctors, nurses, and nutritionists were trained by 16 facilitators who had been trained the week before the course. Of the 40 trained, 30 were from hospitals and 10 were from the Regional Health Office (RHO) and the Integrated Management of Childhood Illness (IMCI) Unit of the Ministry of Health. A ratio of 1 facilitator to 3–6 participants is recommended for the course, but this ratio was reduced to build capacity to deliver future training.

Each hospital team identified a leader, prepared an implementation plan, and began training relevant staff and interns. Scales, stadiometers, and heaters were provided where needed and feed preparation facilities were improved. Attention was given to respecting mothers and involving them in provision of care.

Over 12 months, RHO and MOH staff made 4–5 follow-up visits to each hospital to evaluate progress. Case fatality rates (CFRs) fell progressively during this period. The main causes of death were sepsis and overhydration. Having untrained staff on duty, especially at night, was associated with increased deaths. Acquisition of combined mineral vitamin mix proved difficult until it was included in the national list of essential medicines.

**2006–2012–Expansion phase:** Scaling up training began with tertiary hospitals using the WHO Training Course. In 2007, the mean CFR in 9 tertiary and 3 district hospitals was 25% and after training this fell to 8%. Reimbursement of treatment costs (US$100 per child) was restricted to hospitals that had received WHO-course training and approval of the health insurance system. This initially caused resentment among some untrained hospitals that received a lower reimbursement as their (inappropriate) treatment was less costly.

The severe acute malnutrition (SAM) management protocol was harmonized with the IMCI algorithm, referral and back-referral procedures were defined, and all supplies were included in the national list of essential medicines.

**2012 onward–Consolidation phase:** SAM management has been standardized within the health system and follows international norms. There are fewer cases of SAM; moderate acute malnutrition is treated in primary health facilities, thus halting progression to SAM; poverty and illiteracy rates have decreased; and coverage of water and sanitation has expanded.

In 2019, a Unified Health System (Sistema Unico de Salud) to provide free universal health care was launched. It uses new technology, including teleclinics and mobile phones, to improve access to specialized medical services in rural communities. Management of SAM is part of this scheme.

Integration into the health system, advocacy, induction, supervision, leadership, and teamwork, which were key factors to sustained implementation in South Africa, were also relevant in Bolivia. We discuss some specific factors in Bolivia.

### Key Factor 9: High-Level Team to Link With Health Insurance System

Crucial to implementation was integrating the guidelines into the national public health insurance system. For this, an influential team was needed comprising members of the Bolivian Pediatric Society, the head of the Regional Health Office, and her chief nutritionist. Also crucial were follow-up actions, and the most helpful were the team's reinforcement of training, emphasis on correct chart filling, use of monitoring tools for decision making, and death reviews.

Crucial to Bolivia's implementation process was integrating the guidelines into the national public health insurance system.

### Key Factor 10: Malnutrition Zero Program

An important stimulus in 2007 to scaling up nationwide was the Government's Malnutrition Zero program. One of the program's goals is a CFR of less than 5% for children hospitalized with SAM. Actions included establishing acute malnutrition treatment units in all tertiary hospitals and forming in-service teams to train all health staff in primary, district, and tertiary facilities in the 10 steps. The units also coordinated a network of primary and secondary health care facilities with staff expected to diagnose SAM correctly and refer cases to the hospital, manage moderate acute malnutrition, and take preventive measures.

## MALAWI COUNTRY STUDY

Malawi, with a population of 19 million, is a low-income country. Malawi is landlocked with varied topography, heavily relies on agriculture, is vulnerable to catastrophic floods and droughts, and is characterized by a heavy burden of disease among children and adults. The health system suffers from a lack of resources and a preponderance of staff deployed in urban areas and tertiary hospitals. The Malawi Ministry of Health (MOH) has been addressing the country's malnutrition problem through a multisectoral approach, centered around the integration and scale-up of CMAM into routine health service delivery.^23^^,^^29^ CMAM started in 2002 as operational research in Dowa district with technical support from Concern Worldwide and Valid International during a humanitarian emergency. In 2003, CMAM was extended to Nkhotakota district. In 2004, the MOH added 12 districts in different zones with support from bilateral and United Nations partners. In 2006, the MOH adopted CMAM not only for emergency situations but as a developmental approach.^30^

The Malawi Ministry of Health has been addressing the malnutrition problem using a multisectoral approach that integrates and scales up CMAM into routine health service delivery.

In 2014, at the conclusion of the first 5-year CMAM operational plan, CMAM services had reached about 90% of health facilities. In preparation for the second 5-year plan, a bottleneck analysis was conducted of determinants of CMAM coverage and problems.^31^ This identified inadequate supply of commodities, inactivity of most outreach volunteers, and lack of adequately trained health care staff, resulting in poor outcomes.

In CMAM, children with uncomplicated SAM are treated in the community. Those with medical complications, including those with poor appetite or severe edema, receive inpatient care, which is our article's focus. In 2015, national CMAM data for Malawi indicated that inpatient management met the Sphere standards with an overall CFR of 9.6%. However, 7 district hospitals that had an average CFR of 11.6% failed to reach the Sphere CFR standard of 10.0%.^32^

Box 3 shows the actions taken to improve the quality of inpatient care through training and supportive measures.^32^^–^^34^

BOX 3 **Methods Used to Improve Quality of Inpatient Care for Severe Acute Malnutrition in Malawi**

To improve the quality of care, the Ministry of Health (MOH), with the support of development partners, implemented a series of cascade activities.^32^^,^^33^ In 2016, the MOH updated the national guidelines and severe acute malnutrition (SAM) treatment protocols to align with the latest global guidance and evidence.^34^ In 2017, the regional World Health Organization (WHO) trainer trained a national training team, comprising 8 master trainers, a course director, and clinical director. These master trainers conducted district and facility training for nurses and clinicians using the WHO Training Course. The MOH along with the Paediatric and Child Health Association of Malawi then trained these national-, district-, and facility-level trainers as mentors. Consequently, each of the 104 inpatient facilities has at least 1 mentor. Part of their role is to induct incoming staff and to keep checklists to be used by facility supervisors and managers. The checklists are used to monitor quality of care, help staff to adhere to the 10 steps, and conduct death audits.

During a 17-month period starting in April 2016, quality improvement (QI) efforts were introduced, in which district health management teams and facility-based health care providers identified factors associated with high mortality during inpatient care.^32^^,^^33^ In each hospital, QI teams were formed, comprising clinicians, nurses, health assistants, ward attendants and nutrition assistants to problem solve and coordinate implementation of quality improvement. A death audit team was formed to analyze the cause of death and make recommendations.

Through this QI process, each hospital QI team systematically identifies problems and causes of the high death rate and develops priority solutions to improve case management. Facilities routinely track and analyze cure, death, default, and nonrecovery data, and hospital QI teams track data on initial nutrition and clinical assessment, HIV testing, adherence to the 10 steps, and death audits. Progress in improving quality of care is assessed during mentorship visits by the national team and discussed immediately at the facility. Data are reviewed and discussed at biannual learning sessions, and hospital QI teams share information on changes and solutions that are most effective in improving SAM patient outcomes.

### Impact of Quality Improvement in 7 Hospitals With CFR Above 10%

Table 1 shows the reported improvements in the 5 aspects of SAM management considered most challenging before quality improvement.^32^^,^^33^

**TABLE 1.** Reported Impact of a Quality Improvement Intervention on 5 Aspects of SAM Management in 7 Malawi Hospitals That Had a Case Fatality Rate of More Than 10%

Aspect of SAM Management Targeted	Baseline, %^a^ (January 2016)	After 12 Months, % (February 2017)	After 17 Months, % (August 2017)
Assessing medical complications and nutritional status	23.0	87.0%	94.6
Prevention of dehydration	20.6	96.	95.8
Treatment of dehydration	14.1	89.7	92.9
Immediate cautious feeding	26.7	93.7	94.7
Death audits within 72 hours of occurrence	0	85.0	50.0

Abbreviation: SAM, severe acute malnutrition.

a Percentage of SAM cases.

Despite the reported improvements in care, the CFRs for the 7 hospitals remained above 10%. Death audits indicated that most deaths were due to late presentation, medical complications, mismanagement of emergencies in the outpatient department, and lack of antibiotics—challenges that extend to other parts of the health system.

To date (2021), 104 (more than 95%) health facilities provide inpatient management for children with SAM with an overall CFR of 9.8% in 2019.

### Key Factor 11: Building Confidence Through Post-Training Support

The national team of master trainers, comprising experienced clinicians, nurses, and nutritionists from the MOH, Malawi College of Medicine, and the College of Health Sciences, provided regular post-training support and mentoring to staff at facilities. This motivated staff and built their confidence in managing SAM.

### Key Factor 12: Integration With Emergency Care

Scale-up of a Malawi national ETAT protocol, which includes emergency care of complicated SAM, started in 2012. Implementation was challenging because (1) a long and complicated patient flow in the outpatient department caused treatment delays; (2) outpatient department staff did not have adequate skills to conduct ETAT steps, which resulted in mismanagement of the child's condition; and (3) erratic supply of essential drugs and antibiotics.^32^ This challenge is being resolved through the engagement of the Paediatric and Child Health Association whose team of child health nurses and clinicians provide on-the-job training to health staff to strengthen ETAT at all critical points at facilities. The current coverage of the protocol is 40%.

### Key Factor 13: Staff Rotation and the Need for Pre-Service Training

The policy to rotate clinicians annually, and in some facilities monthly, adversely affects the quality of care. Trained nurses also rotate to other wards. Therefore, induction of incoming staff is vital. Pre-service training provides a sustainable way of ensuring the competence of health care providers. Pre-service training started in 2016 and is currently ongoing in collaboration with the MOH, Malawi College of Medicine, and other partners. Preparatory activities included a review of nursing, midwifery, and medical curricula and updating them to incorporate core nutrition competencies, development of CMAM teaching materials, and building capacity of educators on the new content.

## GHANA COUNTRY STUDY

Ghana is a middle-income country with a population of about 31 million, more than half of whom are urban. The world price for cocoa beans is a major factor in Ghana's economy. There is universal health care through the national health insurance scheme. Health care expenditure is low compared with the regional average (which itself is low). Health delivery is decentralized through the country's 16 regions and 261 metropolitan/municipal/district assemblies. Facilities for treating SAM in Ghana were sparse before 2007 when the MOH and Ghana Health Service adopted CMAM. They envisaged a 2-phased scale-up of CMAM. First, in 2008, outpatient and inpatient health staff and community volunteers were trained in 9 “learning sites” and outpatient facilities were established. Based on lessons learned from these sites, Phase 1 scale-up was initiated in 2009. By the end of Phase 1 in 2012, CMAM training and implementation had extended to 5 of the country's 10 regions, with a focus on strengthening the Ghana Health Service capacities and developing competencies and sustainable services for SAM management.^35^^,^^36^ Partners included the U.S. Agency for International Development, Food and Nutrition Technical Assistance Project, UNICEF, and WHO.

Phase 2 scale-up was planned for 2013–2017. By 2013, CMAM had been initiated in 87 metropolitan/municipal/district assemblies but scaling up was not fully achieved. The bottleneck was government financing, which had an adverse effect on all community-based health services, leading to staff absences at health facilities, lack of medicines and ready-to-use therapeutic food, volunteer fatigue, and low motivation and poor attitudes of staff.^35^^,^^36^

### Implementing eLearning to Improve Management of SAM

In a related endeavor and recognizing that capacity building for CMAM requires an affordable, scalable training solution, in 2012, a small group of educators in Ghana began to use the freely available Malnutrition eLearning course^37^ for pre-service and in-service training. During 2015–2017, the impact of the course on knowledge gained, quality of care, and CFR was evaluated in 9 hospitals and 7 academic institutions across Ghana. Four contextually appropriate delivery models (online, institutional workstation, mobile training center, and mixed) were used. The baseline evaluation found few hospital staff had received any training in SAM management despite being responsible for the care of children with SAM. The course was associated with significant gains in knowledge, understanding, and skills in assessing, diagnosing, and managing SAM, and improvements were observed in almost all components of the WHO 10 steps.^24^ Before training, CFRs ranged from 4.0% to 28.6%. The mean CFR fell significantly from 6.0% pre-training to 2.9% post-training.^25^

The malnutrition eLearning course that educators used in pre-service and in-service training was associated with a significant reduction in mean CFR from 6.0% to 2.9%.

Many who took the course reported that they shared their knowledge with colleagues, mentored other cadres, and used illustrations from the course to counsel mothers and carers. Six months post-training, 2 district hospitals established malnutrition units instead of referring cases to a tertiary hospital. Two hospitals integrated the course into their induction/in-service training and another made it mandatory for incoming staff.

In the academic institutions, course completion rates and knowledge gained were higher where the course was integrated as a required element of the curriculum.^26^ Six institutions integrated the course into teaching within 12 months. Currently, all 7 academic institutions are using the course.

The key factors that were associated with success in South Africa—involvement of hospital managers and district health personnel; data gathering for action and advocacy; building of local capacity for sustainability—also contributed to the success of capacity building through eLearning in Ghana. Induction of incoming doctors and nurses, another key factor, was initiated as a result. Two other key factors in Ghana are training a critical mass and ensuring contextually appropriate delivery.

#### Training a Critical Mass

The malnutrition eLearning course trains users how to assess, classify, and manage SAM in 3 modules, with each taking 2–3 hours to complete. It facilitates self-directed learning enabling many to be trained in a short time, leading quickly to a critical mass of trained hospital personnel with a shared understanding of what to do. This facilitated collaboration and operational changes being driven from the bottom-up, with staff proposing to their managers improved management of SAM.

#### Contextually Appropriate Delivery

Having 4 eLearning delivery models overcame limited information technology infrastructure and provided equivalent learning/training opportunities. The confidence gained in using the Internet for learning enabled users to access other eLearning opportunities.

## CURRENT CHALLENGES IN LMICS

The poor clinical performance of recent graduates in LMICs raises concerns about gaps in knowledge and models of pre-service education.^38^^,^^39^ Medical and nursing curricula rarely include malnutrition leading to a reliance on in-service training. However, the goal must be to realign pre-service training so that it is fit-for-purpose and competency-based for effective practice.

South Africa: Poor internet coverage in rural areas limits the use of online courses; alternative delivery models as in Ghana will be helpful.

Bolivia: Maintaining the quality of service and expertise in managing SAM is challenging, given the reduced number of patients.

Malawi: Despite investment in quality improvement interventions, fidelity to treatment protocols is hampered by intermittent supplies of antibiotics and commodities, lack of basic equipment, and the poor physical conditions of wards, such as having no window panes. This emphasizes the need to create an enabling environment for effective quality improvement implementation.

Ghana: Scaling up CMAM remains a major challenge. A nascent community of practice aims to implement malnutrition eLearning in pre-service and in-service training and build national capacity for scaling up CMAM, but this will require approval by health care training regulatory bodies and Ghana Health Service endorsement.

## DISCUSSION

A considerable proportion of pediatric hospital admissions and deaths in LMICs are young children with SAM^40^ and most of these deaths are avoidable if WHO guidelines are followed. The South Africa, Bolivia, and Malawi country studies provide important evidence that improved inpatient management of SAM is (1) scalable in routine health services, and (2) scalability is achievable within different contexts and health systems. Effectiveness in reducing mortality appears to be retained at scale. These findings are notable as few successful small-scale health interventions ever achieve scale-up.^41^^,^^42^

Implementation science is gaining recognition, and more than 10 frameworks for scaling up have been identified and significant factors described.^41^^,^^43^^,^^44^ Nevertheless there are few real-world accounts of the process from concept of a health intervention to large-scale implementation, especially in LMICs. We consider the strengths of the study to be our mapping of the process in different contexts and identification of key factors (Table 2).

**TABLE 2.** Summary of Key Factors That Facilitated Change in Quality of Care

Participatory ethos and involvement of hospital managers and district health personnel	A participatory approach motivated and enabled staff to implement WHO guidelines, build training teams for rollout, establish standards, and set expectations for improved quality of care.
Data gathering for action and advocacy	Reporting of CFRs at ward and national levels aided monitoring of progress and problem solving. Operational research led to advocacy for guideline adoption and wider actions.
Building of local capacity for sustainability	Collaborations helped build specialist teams to improve staff competencies. Capacity building took many years due to lack of skilled and experienced trainers. eLearning offered an opportunity to build local capacity quickly.
Induction of incoming doctors and nurses	In-service training of new staff and those on rotation was essential to deal with inadequacies in pre-service medical and nurse training.
Triage and emergency care	Triage and timely treatment reduced early deaths. Emergency care was adjusted to allow for the physiological/metabolic changes that exist in SAM.
Supervision, leadership, teamwork, and post-training support	Supportive supervision on the ward, post-training mentoring of staff, good leadership, and teamwork built confidence, raised morale and job satisfaction, and helped sustain improved quality of care.
Keeping it short and simple	Identifying memorable key messages aided guideline adherence. Wall charts served as reminders, and job aids reduced errors.
Political commitment and administrative policies for sustainability	Implementing and sustaining WHO guidelines at scale required ministerial support, regulatory and administrative policies, strategic planning at provincial and district levels, and budgetary provision.
Partnerships	Partnerships and collaborations aided credibility, operational research, capacity building, and technical and financial support. Short term financial support hindered sustainability.

Abbreviations: CFR, case fatality rate; SAM, severe acute malnutrition; WHO, World Health Organization.

South Africa dominates the case studies, partly because implementation research funding was received from the Health Systems Trust and WHO and the focus was on inpatient management in contrast to Malawi and Ghana where the focus was on community-based management. Although the successful country studies we included traversed different scale-up pathways, they share certain features, some of which we discuss here.

### Political Commitment and Strategic Planning

Scaling up was aided and energized by a commitment to the Millennium Development Goals, which included a commitment to reduce child mortality. In South Africa, the work in Eastern Cape informed how the national and provincial scale-up strategy would be designed. Scaling up accelerated after 2011 when SAM management became part of primary health care re-engineering policy with ministerial support, underpinned by the national development plan. Regulatory and administrative policies followed, including national SAM treatment guidelines in 2015 and standards for local commercial production under license of therapeutic feeds F75 and F100. The national Department of Health developed, coordinated, obtained funding from implementing partners (U.S. Agency for International Development and FHI360), and worked with provincial and district management teams to roll out targeted training of district hospital staff and management teams.

Scale-up in all 4 countries was facilitated by national commitments and policy changes.

In Bolivia, SAM management has strong political commitment, which enabled its inclusion into the national public health insurance system, and it continues to be an integral part of health policy. Central planning started in the expansion phase (2006–2012) and was one of the 4 lines of action in the Malnutrition Zero program and continued thereafter.

In Malawi, CMAM was directed, monitored, and coordinated in the Office of the President and Cabinet and implemented as part of a primary health care package. From 2006, CMAM was a component of national nutrition policy, with national guidelines, standards, targets, monitoring tools, training manual, and nutrition resource kit. Various committees guided scale-up and quality implementation.

In Ghana, CMAM was included in national nutrition strategic plans. Initial implementation was good with more than 1,000 trained with the WHO training course. However, procurement of commodities and capacity building of health personnel were donor dependent and short term. Without sufficient government commitment of resources, effective integration of CMAM into the health system was not sustained—a problem mirrored in Burkina Faso.^45^ Short-term, donor-driven health programs often fail to scale up even when successful.^42^^,^^46^

### Approach to Scale-Up

In South Africa, initially scaling up occurred organically when funding and training capacity permitted. In Bolivia, the Malnutrition Zero program provided a more formal structure for scaling up. At the launch, a package of measures for improving inpatient management of SAM was already in place. These measures included a case-management protocol; a tried-and-tested training course; and a training hub in 2 hospitals with previously trained, proficient pediatricians, nurses, and nutritionists who had experience delivering the WHO training course.

Malawi and Ghana had scale-up plans for CMAM from the outset, although plans were only partially completed in Ghana. The 7 attributes most likely to be associated with successful transfer of innovations to scale^47^ are that the innovation: (1) is credible, (2) is observable, (3) is relevant for the context, (4) has a relative advantage over existing practices, (5) is easy to implement, (6) is compatible with the values and needs of users, and (7) is testable in the context. These attributes were manifest in the country studies. In Ghana, the donor-driven CMAM program achieved partial scale-up in a relatively short time, and one may consider whether longer-term donor support, as in Malawi, would have been preferable. On the other hand, Ghana is economically stronger than Malawi and an expectation for a greater commitment by Government to increased spending on health may be reasonable.

### Partnership of Organizations

Research-stakeholder partnerships can bring credibility, innovation, and a contextual framework to scaling up.^43^ Partnerships and collaborations were important in all 4 country studies. South Africa and Bolivia had strong university-health system partnerships, which led to operational research, capacity building, and advocacy that formed the foundation for scale-up. The London School of Hygiene & Tropical Medicine assisted with training and served as a conduit for collaboration with WHO for implementation research in South Africa and technical support in Bolivia, including technical support for establishing the Malnutrition Zero program. Additionally, in Bolivia, from 2006, the lead trainer and scale-up organizer and the influential Bolivian Pediatric Society had direct access to the leadership of the supportive MOH and to the National Council for Food and Nutrition (Consejo Nacional de Alimentacíón y Nutrición*)*, headed by the President. Malawi had considerable external support, both technical and financial, and similarly benefited from university-stakeholder partnership, including research into local production of ready-to-use therapeutic food for which Malawi is now 90% self-sufficient. In Ghana, the CMAM program had less strong partnerships with universities and was unable to break away from donor dependency. In contrast, capacity building through the eLearning course had strong partnerships between the University of Southampton and the Kwame Nkrumah University of Science and Technology, and between Kwame Nkrumah University of Science and Technology and the pre-service and in-service health providers who participated in the study.

### Long Duration of Scale-Up

The time required for scale-up is a notable feature of these country studies. Another notable feature is the unevenness of the process from location to location and with time in any one location. The opportunity for scale-up was built from very different starting conditions in each situation. However, all 4 countries started from a very small base, which was advantageous as time was necessary to demonstrate success convincingly; unite opinion as to the value of implementing the guidelines; advocate adoption; develop agility in problem solving; and build training capacity, support systems, and leadership. Each eventually reached the position where it could be considered that the factors identified as being important in the different frameworks for scale-up were met, but it was not possible to identify or predict in which order these were formally recognized, acknowledged, and addressed. Scaling up health interventions is complex with multiple stages,^46^ and a duration of 15 years is not unusual. In South Africa, national scale-up was largely achieved during 6 years, between 2011 and 2017, but the groundwork was built up over a much longer period (12 years). In Bolivia, scale-up of 10 years would have been longer had it not been for the timely launch of the Malnutrition Zero program. Resource allocation is likely to influence the speed, fullness, and quality of scale-up. The respective health expenditures in 2010 for South Africa, Bolivia, Malawi, and Ghana were US$635, US$102, US$35, and US$74 per caput.^48^ Although influenced by several factors, common to each situation was the persistence of a relatively senior person(s) in actively seeking solutions to identifiable, clearly defined problems and grasping opportunities as they arose. In the future, durations of scale-up may be shorter as new opportunities exist such as eLearning, which can accelerate capacity building.

Scaling up health interventions is complex with multiple stages, and a duration of 15 years is not unusual.

### Alignment With WHO Guidelines

National guidelines in all 4 countries parallel those of WHO. The 10 steps are relatively straightforward and easily understood, which facilitated adoption and scale-up. The CFR reflects quality of care, and the crux of low CFRs is doing simple things well, including basic nursing tasks to aid decision making. Fidelity to WHO guidelines is variable. Diligence is improved when staff understand the purpose of each task. Otherwise, staff may skip tasks or even consider them oppressive, especially if they are repetitive. We have found that both face-to-face training and eLearning enable understanding of the importance of fidelity and attention to detail. Fidelity also depends on staffing levels; availability of antibiotics and other supplies; and the quality of induction, supervision, and leadership. Mentoring and supportive supervision provide many opportunities for improving adherence through positive feedback, problem solving, and developing relationships that raise morale and job satisfaction. Participation in practice drills within ongoing skill training and rewarding participation with continuing professional development points has incentivized improved quality of obstetric care in South Africa^49^ and might be applicable to improving the fidelity of SAM management guidelines.

Loss of trained pediatric staff to better-resourced private health and nongovernmental organization sectors hampered continuity of expertise in the African country studies and added to the shortage of competent staff. Although commercial production of therapeutic feeds has simplified routines, supplies in Malawi can be intermittent. In Bolivia, affordable and reliable stocks of F75 and F100 are prepared at each hospital from milk, sugar, oil, and an electrolyte/mineral solution.

### Capacity-Building Approaches

Capacity building is at the heart of the successful country studies and was a limiting factor in scaling up CMAM in Phase 2 in Ghana. The 6-day WHO training course provides in-depth knowledge and skills with supervised practice and can generate a nucleus of proficient trainers. Recently in South Africa, a trial was conducted of a modular training program delivered in 2-hour interactive sessions to small groups to improve clinical care.^18^ By repeating a session 4 times in a single visit, it proved possible to reach all staff on day and night shifts. The Internet has the potential to help reduce inequalities in training opportunities between wealthy countries and LMICs. Where face-to-face training is limited, especially through lack of experienced trainers, eLearning can offer a scalable approach to build capacity quickly and improve competencies and can free up trainers to deliver supervisory and other support activities. Delivering eLearning is less costly than face-to-face teaching.^26^ Whichever approach is chosen, training must be integrated within routine government programs as it is important in designs for implementation, sustainability, and scale-up.

### Data Systems

For effective scale-up, information systems are needed to assess the progress of implementation and identify potential problems and solutions. In the successful country studies, comprehensive data-gathering systems were linked to strategic planning and management. In South Africa, during the roll-out, all 242 district hospitals were gradually inducted to begin reporting SAM admissions and deaths. In Bolivia, before 2006, the national health monitoring system (Sistema Nacional de Información en Salud) reported weight-for-age. After this date, height-for-age and weight-for-height indicators were added to monitor stunting and wasting, respectively.

### Limitations

A limitation of this study could be including just 4 countries. At the outset, we approached all contacts who might know of scaled-up programs. We followed up all those country programs identified, but none proved to be scaled up countrywide. As far as we know South Africa, Bolivia, and Malawi are the only programs where SAM case management guidelines are implemented on a national scale and have become part of national plans and policy, although omission of other examples remains a possibility.

## CONCLUSION

The experience reported here indicates that the successful adoption of guidelines requires sensitivity to the varying character of different contexts. Sustained change requires not only the adoption of the guidelines that capture best practice but also a system-wide shift in the approach to the delivery of care. Frontline staff have to be confident in their ability to deliver appropriate care with competence. In addition, they have to be enabled to achieve the high standards of care that can only be brought about by providers and managers adopting policies and practices that support the necessary changes.^12^^,^^50^ The common factor across each example was the ambition to support change and the willingness to adopt and accommodate policies that strengthened and reinforced an environment within which the guidelines could be securely established as usual practice.

The 2030 Sustainable Development Goals provide an opportunity to commit to scaling up effective child health interventions. We know what to do.^5^^,^^6^ The challenge is how to implement at scale. Clearly, there needs to be political commitment and an enabling environment with leadership at all levels, from policy makers within ministries of health to ward managers. Good facility-based care underpins effective CMAM. In the short term, in-service training will remain the main channel for delivering the knowledge and skills needed, but pre-service training must be reformed.

The 10 steps, adopted by the United Nations system and representing the underlying principles of care, have stood the test of time. While having universal application, understanding how best to adopt and apply these principles requires an ability to (1) identify context-specific problems; (2) seek an effective solution that is feasible and sensitive to local circumstances; and (3) build a workforce that is competent, reflective, respectful, confident, encouraged, and supported. Attaining these abilities can be a challenge but, with guidance and teamwork, success can have a multiplier effect. Future studies might usefully address how best to engage policy makers, health professionals, and their national societies in bringing together all the talent available and establishing alliances such as communities of practice, clinical specialist teams, and clinical information networks^51^^,^^52^ to implement and scale up WHO malnutrition treatment guidelines and use new opportunities such as eLearning to build workforce capacity.

## Translation

**En Español**

**Ampliación a Escala del Manejo Mejorado de la Desnutrición Aguda Grave Para Pacientes Hospitalizados: Factores Clave y Experiencias de Sudáfrica, Bolivia, Malawi, y Ghana**

**Principales hallazgos**

Reportamos las lecciones aprendidas y el abordaje utilizado para la implementación a escala de la “Guía de manejo intrahospitalario del desnutrido agudo grave” de la OMS en los servicios habituales de salud en 4 países.

Se muestra una avance sustancial en la supervivencia de niños con desnutrición aguda grave a pocos meses después de la implementación de la guía, aunque la adquisición de practicas de atención adecuadas, tomo varios años al ampliar esta atención a escala nacional.

Demostramos que la guía de la OMS se puede ampliar a nivel nacional y que su efectividad en la mejora de la supervivencia parece mantenerse al incrementar este manejo a escala. Las características clave para el éxito incluyen: colaboración para mejorar la capacidad y realizar actividades de investigación operativa y abogacía para la adopción de las guías; equipos de especialistas para tutoría y la creación de confianza y competencias a través de una supervisión que brinda apoyo; compromiso político y políticas administrativas para la sostenibilidad.

**Implicaciones principales**

Responsables de políticas de salud, profesionales de salud, y sociedades científicas nacionales deben brindar en conjunto todo el talento disponible y establecer alianzas como comunidades de practica para implementar la “Guía de manejo del desnutrido agudo grave”, ampliarla a escala nacional, y utilizar oportunidades como la enseñanza en línea para mejorar la capacidad del equipo de trabajo.

Educadores de salud deben revisar la curricula de enseñanza de pregrado de medicina y enfermería; y alinearla a los objetivos de la “Guía de manejo intrahospitalario del desnutrido agudo grave” a través de una practica efectiva basada en competencias.

**Resumen**

La desnutrición aguda grave (DAG) puede causar una elevada mortalidad, especialmente en niños muy enfermos tratados en hospitales. Varias escuelas de medicina y enfermería no enseñan adecuadamente el manejo de la DAG, o simplemente lo omiten. Hay escasez de profesionales y docentes con experiencia en este manejo que sirvan como ejemplo de buenas prácticas o que participen en el desarrollo de capacidades. Consideramos estudios de caso de cuatro países donde se realizo la ampliación a escala de la “Guía de manejo intrahospitalario del desnutrido agudo grave” de la Organización Mundial de la Salud (OMS); para mejorar el manejo de la DAG en pacientes hospitalizados que asisten a servicios de escasos recursos del sector público en Sudáfrica, Bolivia, Malawi y Ghana. Basándonos en informes de implementación, datos cualitativos y cuantitativos de nuestras investigaciones, recopilación de datos prospectivos y retrospectivos, autorreflexión y experiencias compartidas, revisamos nuestros enfoques de desarrollo de capacidades para mejorar la calidad de la atención, la efectividad de la implementación y las lecciones aprendidas. Los estudios reportados proporcionan evidencia importante que muestra que el manejo mejorado de la DAG en pacientes hospitalizados se puede ampliar a los servicios habituales de salud a escala nacional en diferentes contextos y sistemas de salud. La eficacia en la reducción de las muertes por DAG en pacientes hospitalizados parece mantenerse en la implementación a gran escala.

Los estudios de país muestran evidencia del impacto sobre la mortalidad en las primeras etapas de implementación, y durante el proceso de ampliación. Sin embargo, se necesitaron muchos años para desarrollar la capacidad de la fuerza laboral, establecer procedimientos de supervisión y tutoría e institucionalizar las pautas dentro de los sistemas de salud. Las características clave para el éxito incluyeron: colaboración para desarrollar capacidades, realización investigaciones operativas y promoción para la adopción de directrices; equipos de especialistas para asesorar y generar confianza y competencia a través de una supervisión que brinda apoyo y; compromiso político y políticas administrativas para la sostenibilidad. Para que el personal de primera línea confíe en su propia capacidad para brindar la atención adecuada de manera competente, se necesita un entorno propicio, políticas y procesos de apoyo en todos los niveles del sistema de salud.

**Peer Reviewed**

**First published online:** March 22, 2022.
